# Use of Surface Enhanced Blocking (SEB) Electrodes for Microbial Cell Lysis in Flow-Through Devices

**DOI:** 10.1371/journal.pone.0102707

**Published:** 2014-07-17

**Authors:** Abdossamad Talebpour, Robert Maaskant, Aye Aye Khine, Tino Alavie

**Affiliations:** Qvella Incorporation, Richmond Hill, Ontario, Canada; University of Florida College of Medicine, United States of America

## Abstract

By simultaneously subjecting microbial cells to high amplitude pulsed electric fields and flash heating of the cell suspension fluid, effective release of intracellular contents was achieved. The synergistic effect of the applied electric field and elevated temperature on cell lysis in a flow-through device was demonstrated for Gram-negative and Gram-positive bacteria, and *Mycobacterium* species. The resulting lysate is suitable for downstream nucleic acid amplification and detection without requiring further preparation. The lysis chamber employs surface enhanced blocking electrodes which possess an etched micro-structured surface and a thin layer of dielectric metal oxide which provides a large effective area and blocks transmission of electrical current. The surface enhanced blocking electrodes enable simultaneous suppression of the rapid onset of electric field screening in the bulk of the cell suspension medium and avoidance of undesired electrochemical processes at the electrode-electrolyte interface. In addition the blocking layer ensures the robustness of the cell lysis device in applications involving prolonged flow-through processing of the microbial cells.

## Introduction

The unprecedented advances in detecting and identifying microorganisms using nucleic acids have not been adequately matched with corresponding progress in pre-analytical sample preparation techniques required for efficiently and rapidly providing an inhibitor and contamination free nucleic acid suspension. Consequently, despite its specificity and sensitivity, polymerase chain reaction (PCR) has not replaced the much slower standard microbial culture-based techniques as the frontline diagnostic test in the clinical microbiology laboratory. For microfluidic applications, often favoured by developers of automated molecular based platforms, the difficulties are enhanced in the adaptation of traditional cell lysis techniques such as mechanical, chemical and enzymatic lysis methods. These generally require the addition of reagents for the lysis step and some form of extraction and purification of the target nucleic acids.

Over a decade ago, seemingly inspired by the extensive studies on microorganism inactivation by pulsed electric fields (PEF) [1], irreversible electroporation was suggested as a convenient method of microbial cell lysis for molecular assay chips [2]–[3]. This approach, involving the formation of electrically induced nanoscale pores in the cell membrane, has been successfully demonstrated for the lysis of mammalian cells and a range of microbial species [4]–[6]. In many previous studies involving the application of electric fields, the term “lysis” has been used to refer to both the inactivation of microbial cells and the permanent rupturing of the cell wall. However, a careful review of the literature shows that in most cases, lysis efficiency has only been indirectly inferred based on microbial cell survival rates and not based on direct evidence of the release of intracellular contents such as nucleic acids. The use of indirect metrics for inferring microbial cell lysis can lead to misinterpretation of the effect of the electric fields on the microbial cells because, even though a microbial cell may be rendered inactive by the application of an electric field, it may not be sufficiently lysed to achieve release of nucleic acids.

According to a recent report, the thickness of a cell wall may serve as a barrier against the formation of large pores which are necessary for the release of larger cell contents such as ribosomes [7]. This may be the reason why only a limited number of studies have reported the success of electrical lysis methods to release nucleic acids [2], [4], and success in this regard is restricted generally to Gram-negative bacteria such as *E. coli*. This may also be the reason why electrical lysis methods for efficient release of nucleic acid contents have not yet been demonstrated for microorganisms with bilayer membranes surrounded by a tough cell wall, as in the case of Gram-positive bacteria and fungi.

An alternative approach for achieving reagent-free inline microbial cell lysis is thermal lysis which employs external heating of capillary channels. A recent study [8] has concluded that thermal lysis of *E. coli* cells achieves DNA yields similar to lysing by bead beating only for heating times greater than 10 minutes. This may not be fast enough for typical applications and furthermore the method has not been demonstrated for bacteria with tougher cell walls. Therefore, a platform based on heating alone is not expected to offer a rapid cell lysing module for implementation on microfluidic devices.

In this article, a novel hybrid approach is described that employs both electrical and thermal lysis mechanisms to achieve effective lysis and release of intracellular contents for a wide range of bacterial cells types, including Gram-positive and Gram-negative bacterial and *Mycobacterium* microbial cells. The method involves exposing microbial cells to relatively strong electrical fields in the presence of rapidly increasing temperatures that arise during the application of the electric field. This electrical lysis method, which is termed “E-Lysis” in this manuscript, utilizes an applied electric field to generate a high cellular trans-membrane voltage while simultaneously inducing flash heating due to Joule heating from the ionic current in the cell suspension fluid, and the combined effect of the electric field and the flash heating results in the lysis of the suspended cells. A phenomenological rationale for performing electrical lysis at elevated temperatures is provided by the phase transition model of electro-permeabilization in an external electric field [9]. According to this model, bilayer membranes have several stable states each corresponding to a local minimum of the molecular free energy. At a sufficiently elevated temperature the local minima vanish and the membrane dissolves in the surrounding water forming small micelles. Similarly, in a strong enough external field the free energy minima cease to exist leading to the breakdown of the membrane. Therefore, if a trans-membrane voltage is applied to cells suspended in a high temperature medium, the electric field thresholds needed for the disintegration of the cell membrane and subsequent release of the macromolecules can be substantially lowered [9].

The E-Lysis method was implemented by exposing microbial cells to relatively high electric fields in a microfluidic channel and applying a train of bipolar square pulses through “surface enhanced blocking electrodes”. These electrodes have a finely micro-structured surface on which a thin dielectric layer is formed. It is demonstrated that this approach enables the design of a robust microbial cell lysis device capable of exploiting the synergistic effects of electrical field and heat for efficient release of nucleic acid contents from the cells and delivering assay-ready lysate for performing downstream nucleic acid assays.

## Materials and Methods

### Fabrication of surface enhanced blocking (SEB) electrodes

**Figure 1:**
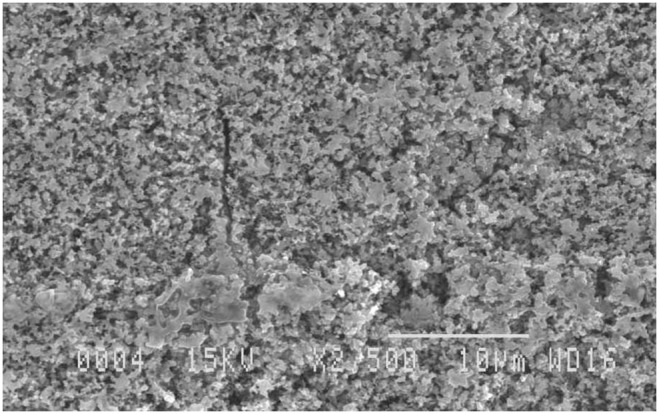
SEM image of the electrode surface. The microstructure profile of Al_2_O_3_ layer on aluminium surface, created by etching and then anodizing an aluminium foil, was revealed by an SEM image with 2500x magnification.

The SEB electrodes are fabricated by electrochemical etching of the surface of 100 µm thick aluminium foils in an aqueous solution containing hydrogen chloride and appropriate additives referring to the recipes disclosed in the US patent 4,276,129 [10]. The etched foils are then anodized in an aqueous electrolytic solution containing boric acid and ammonium borate and a formation current of 0.05 mA/cm^2^ at 100 V [11]. A scanning electron microscopy (SEM) picture of the electrode surface, presented in Figure 1, portrays the enhanced oxidized surface of the aluminium electrode.

### Fabrication of cell lysis device

**Figure 2:**
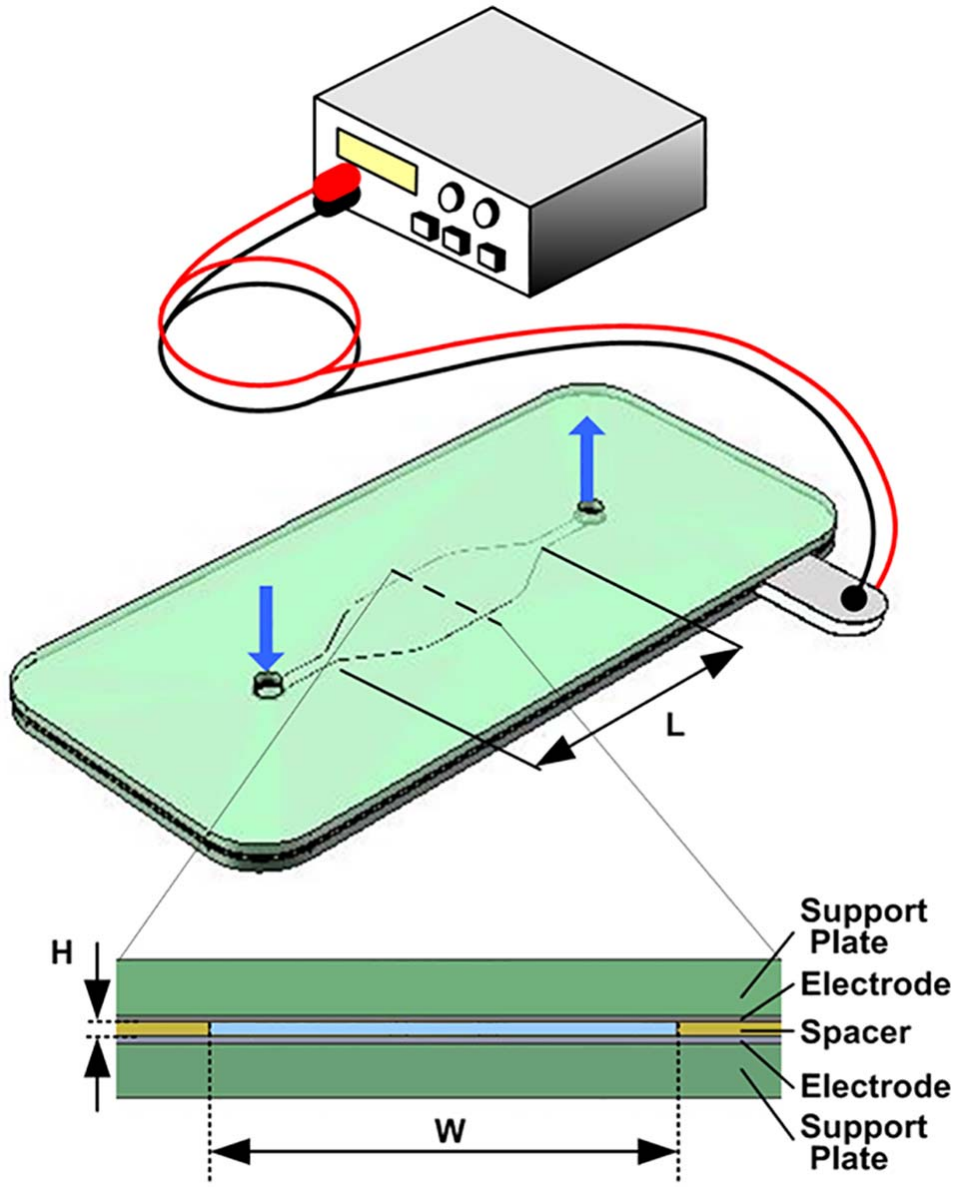
The schematic view of the flow-through electrical lysis device. A microfluidic chamber was fabricated by bonding the electrodes to two flat Lexan plates and then sandwiching a dielectric spacer element between the electrodes. The electronic system supplies bipolar square electric pulses whose amplitude or frequency can be controlled in response to chamber temperature obtained in real-time from chamber conductivity measurements.

A microfluidic lysis device was fabricated by bonding the electrodes to two flat Lexan plates and then sandwiching a dielectric spacer element between the electrodes. The spacer serves to define the side walls of the electrical chamber, provides the fluid seal, and electrically insulates the electrodes from each other. Figure 2 shows an illustration of the chamber device and voltage controller. Two types of electrical chambers were constructed. The standard electrical chamber used in lysis testing had dimensions of 15 mm(L)×6.4 mm(W)×0.2 mm(H). For electrical characterization of the SEB electrodes an electrical chamber with the dimensions of 28 mm(L)×3.2 mm(W)×0.1 mm(H) was used. In this case the smaller height allowed the application of electrical fields with twice higher amplitudes.

### Cell culture

Each single colony of Gram-negative bacteria (*Escherichia coli, Klebsiella pneumonia, Pseudomonas aeruginosa, Enterobacter cloacae and Acinetobacter baumanii*) grown on LB agar (G401, Hardy Diagnostics) was respectively cultured in LB broth (CG51, Hardy Diagnostics) overnight at 37°C. The cells were centrifuged at 7000 rpm for 5 min. The cell pellet was washed twice and re-suspended in 0.1 to 0.5 mM pre-filtered sodium phosphate buffer pH 7.4.

Each single colony of Gram-positive bacterial cells (*Streptococcus pneumonia, Streptococcus sanguis, Streptococcus pyogenes, Staphylococcus aureus and Enterococcus faecalis*) grown on Trypticase Soy agar with 5% sheep blood (A10, Hardy Diagnostics) was respectively cultured in Tryptic Soy broth (U63, Hardy Diagnostics) overnight at 37°C. The cultured cells were centrifuged at 10000 rpm for 5 min. The cell pellet was washed twice and re-suspended in 0.5 mM pre-filtered sodium phosphate buffer pH 7.4.

A single colony of acid-fast bacteria, *Mycobacterium smegmatis*, was grown with agitation for 2–3 days at 37°C in 5 ml of Middlebrook 7H9 (VWR,), containing sterile albumin dextrose complex (ADC) (0.5% BSA, 0.2% glucose, 3 µg of catalase per ml) supplement as well as Tween 90 (0.05%). The cultured cells were centrifuged at 10000 rpm for 5 min. The cell pellet was washed twice and re-suspended in 0.5 mM pre-filtered sodium phosphate buffer pH 7.4.

The concentrations of the bacterial suspensions were estimated by optical density measurement at 600 nm in Bio-Rad SmartSpec 3000 spectrometer, using sodium phosphate buffer as a blank and the conversion factor of 1 OD = 5×10^8^ cells/mL. The bacterial suspensions for the respective experiments were prepared at a concentration of 1×10^9^ CFU/mL, 5×10^8^ CFU/mL or 200 CFU/mL in sodium phosphate buffer, as indicated in respective experiments. The high concentration cell suspension was used for verification of verification of lysis efficiency with the Bradford protein assay and the low concentration cell suspension was used for testing with the real-time reverse transcription PCR assay.

### Cell lysis

Electrical lysis was performed by passing a 60 µL aliquot of cell suspension through the electrical lysis chamber in increments of 10 µL and applying a voltage pulse train at each increment. The voltage pulse train consisted of bipolar square pulses which, prior to initialisation of feedback control, had amplitude of 195 V and a frequency of 10 kHz.

In some experiments, cells were also subjected to mechanical lysis using glass beads for the purpose of comparison. To perform glass bead lysis (GB), an equal volume of glass beads (<106 µm, G4649 Sigma) was added to 60 µL aliquot of cell suspension and mechanically lysed by vortexing at a high speed for 5 minutes. The GB cell lysate was centrifuged at 14,000 rpm for 1 minute to separate the beads and the supernatant was collected for the assay.

### Bradford protein assay

Total protein released into the cell lysate was assayed using Bradford Reagent (B-6916, Sigma). The cell lysate was centrifuged at 7000 rpm for 5 minutes and 50 µL of the supernatant was collected and mixed with an equal volume of Bradford protein assay reagent. The protein concentration was determined by measuring light absorbance at 595 nm and referring to a dose response curve previously prepared by running the Bradford assay on different concentrations of Bovine Serum Albumin (BSA).

### Real time RT-PCR assay

A real-time reverse transcription PCR (RT-PCR) assay was performed to detect a specific target region in 16S rRNA of the bacteria. The primers were designed by sequence alignment software (Bioedit, Ibis Biosciences, USA) and primer design software (Primer3, National Institute of Health). RT- PCR reaction volume of 20 µL was prepared by mixing 5 µL of lysate sample, 10 µL of KAPA2G Robust HotStart 2X PCR reaction mix (kk5515, Kapa Biosystems), 1.2 µL of GoScript reverse transcriptase (A5004 Promega), 1 µL of forward primer (10 µM), 1 µL of reverse primer (10 µM), 1 µl of 100 nM SYTO-9 (S34854, Invitrogen) and 0.8 µL of RNAase free water. One-step real time RT- PCR was performed by reverse transcription at 55°C for 5 min, and activation of HotStart DNA polymerase and inactivation of reverse transcriptase at 95°C for 2 min, followed by 35 cycles of cDNA amplification (denaturation at 95°C for 3 sec, annealing at 63°C for 3 sec, and extension at 72°C for 3 sec) in an Eco Real Time PCR system (Illumina).

For the detection of Gram-negative bacteria, a forward primer 5′-GTTACCCGCAGAAGAAGCACCG-3′ and a reverse primer, 5′-ATGCAGTTCCCAGGTTGAGCC-3′ were used to amplify the 16S rRNA gene fragment of 151 base pairs at a hypervariable region of all Gram-negative bacterial species (nucleotides 484 to 635 using *Escherichia coli* IaI1 as a reference).

For the detection of Gram-positive bacteria, a forward primer 5′-GACAGGTGGTGCATGGTTGTC-3′ and a reverse primer, 5′-ACGTCATCCCCACCTTCCTC-3′ were used to amplify the 16S rRNA gene fragment of 170 bases pair at a hypervariable region of all Gram-positive bacterial species (nucleotides 1035 to 1185 using *Streptococcus pneumoniae G54* as a reference).

For the detection of *Mycobacterium smegmatis*, a forward primer 5′-CCACACTGGGACTGAGATACGGC-3′ and a reverse primer, 5′- CGTATCGCCCGCACGCTCAC-3′ were used to amplify the 16S rRNA gene fragment of 202 base pairs at a hypervariable region of all *Mycobacterium* species (nucleotides 333 to 673 using *Mycobacterium bovis* BCG M140 as a reference).

## Results and Discussion

### Electrical characteristics of the lysis device

The E-Lysis method is performed using a fluidic device having a chamber formed between two opposing electrodes. During the process, electrochemical reactions can take place at the electrode-electrolyte interface, by products of which can create undesirable effects for the application of microfluidic reagent-free cell lysis. For example, gas generation and bubble formation due to electrolysis of the cell suspension fluid [12] can obstruct the fluidic channel. In addition, the products of redox reactions at the electrode interface may damage the electrodes, and degrade target biological molecules in the resulting lysate. In addition, the redox reaction may drastically change the ionic composition of the lysate rendering the liquid unsuitable for the downstream assays without further processing.

The electrolysis products can be partially avoided by operating the device with a high frequency bipolar pulse train. The ions produced at the electrode-electrolyte interface are then significantly neutralized in alternating cycles before diffusing away into the bulk medium [12]. The frequency should be chosen in consideration of the ionic strength of the chamber fluid so as to maximise the effective duration of the applied electric field, which results in higher electroporation efficiency [1].

The potential degradation of target molecules at the interfacial zone by the electrolysis products can be alleviated by providing the electrode surface with a protective permeation layer [2]. Such layers allow the movement of solute ions to the electrode while preventing the macromolecules from reaching the electrode-electrolyte interface. This approach, while reducing ill effects of electrolysis to some extent, does not prevent it entirely and the fabrication of the layer is a difficult multistep process. A preferred method for countering interfacial reactions is insulating the electrodes from the electrolyte with a thin layer of dielectric coating thus forming “blocking electrodes” which avoid direct electrical contact with the liquid within the chamber. However, the presence of the dielectric layer introduces an electrode surface capacitance which accelerates the formation of electric double layers in the ionic solutions near the electrodes and, consequently, screens the bulk fluid in the chamber from the applied electric field within a so-called charging time [13]. Accordingly, the effective electric field experienced by the suspended cells may drop to a small fraction of the nominally applied electric field shortly after the field is applied [14]. This problem can be circumvented by forming the dielectric layer on a conductive substrate with a finely micro-structured surface which substantially increases the surface area of the electrode. The large capacitance that is thereby achieved enables a charging time close to that of nominally smooth non-blocking electrodes, while concurrently avoiding the generation of electrolysis products. This type of electrode is henceforth referred to as a surface enhanced blocking (SEB) electrode.

**Figure 3:**
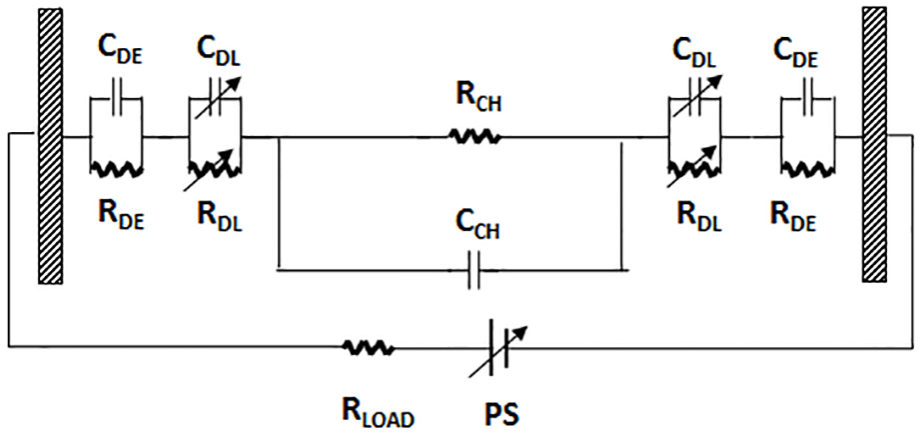
The equivalent circuit model for the electrical lysis device. The electrical lysis chamber modelled by an equivalent electrical circuit following the suggestions of reference [15].

The electrical characteristics of the chamber can be modelled by the equivalent electrical circuit presented in Figure 3 [15]. The capacitance C_DL_ corresponds to the dynamic double-layer capacitance near the interface of dielectric layer and the liquid in the chamber. R_DL_ is the parallel (in the direction of the chamber thickness) resistance corresponding to leakage current in the double layer. In general, values of C_DL_ for flat metal electrode surfaces fall in the range 5–50 µF/cm^2^ depending on the type of electrode, ionic strength and composition of the solution, temperature and voltage [16]. However, electrode surface enhancement will increase this capacitance to higher values. Capacitance C_DE_ is the capacitance of the dielectric layer whose value depends on the layer thickness and the effective area of the electrode. For the electrodes used in the experiments described here, based on the empirically established relation between capacitance and the formation current and voltage [11], C_DE_ is estimated to be about 3 µF/cm^2^. Resistance R_DE_ is the equivalent parallel resistance of the dielectric layer and accounts for leakage current in the capacitor. This resistance is in the order of 10 MΩ due to very low conductivity of the Al_2_O_3_ layer. R_CH_ represents the bulk solution resistance and C_CH_ the bulk capacitance. The value of the bulk solution capacitance C_CH_ = εε_0_ *WL*/*H*, where ε = 80 is the dielectric constant of water, is estimated from the geometry of the chamber to be on the order of 1 pF. This value is so small that it can be approximated by an open circuit. R_LOAD_ is the sum of the power supply output resistance and the input resistance of the electrodes.

**Figure 4:**
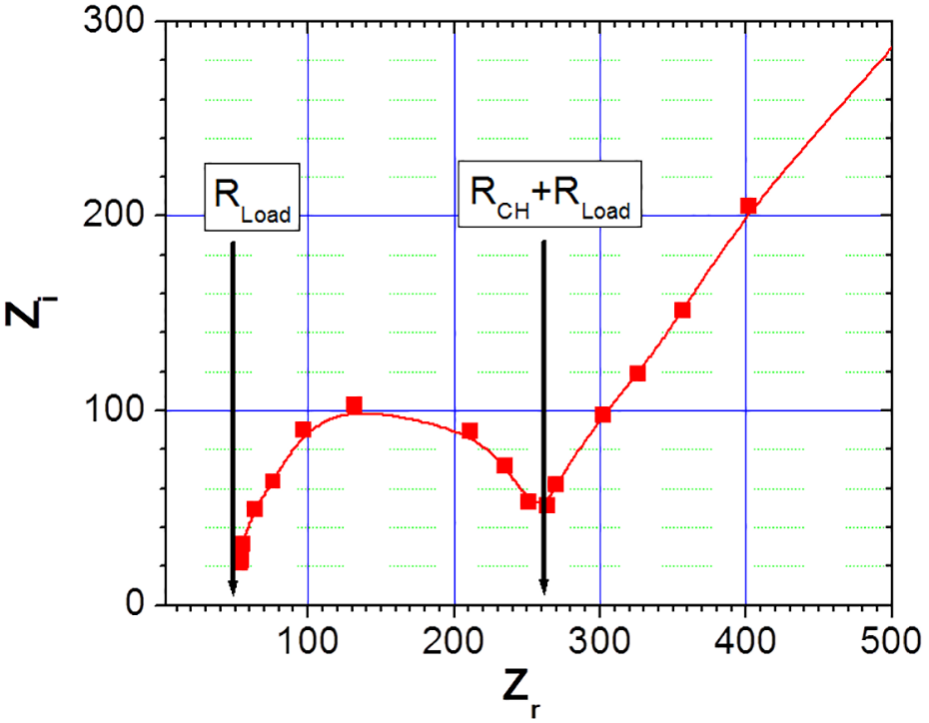
The impedance spectrum of the electrical lysis chamber. An electrical lysis chamber, with dimensions 28(L)×6.4 mm(W)×0.1 mm(H), was filled with 0.25 mM NaCl solution and its impedance spectrum was recorded in the 2 kHz to 4 MHz frequency range.

For the purposes of electrical characterisation the chamber of dimensions 28 mm(L)×6.4 mm(W)×0.1 mm(H) was filled with 0.25 mM NaCl solution. The resistance R_CH_ is estimated to be 220 Ω for this chamber configuration based on the fluid conductivity reported by McClesky [17]. Impedance spectrum measurements were obtained in the frequency range of 2 kHz to 4 MHz, and are presented in Figure 4. The chamber resistance, estimated from these results [18], gives a value of 210 Ω which agrees well with the estimated value. All the electrical parameter values, with the exception of R_LOAD_, R_DE_ and C_DE_ are dependent on the ionic strength of the carrier solution. The load resistance modifies the voltage division among the circuit components and is more important at higher ionic strengths.

The electrical response of chambers can be investigated by adopting the one dimensional model described by Bazant *et al* [14] for which a dilute 1∶1 electrolyte such as NaCl is suddenly subjected to a DC voltage, *V*_A_, by bare parallel-plate electrodes separated by a distance *H*. Due to the build-up of a double layer at the electrode-electrolyte interface the bulk electric field, *E = V_A_/H*, starts to lose uniformity in the thickness (*H*) direction. Ions migrate in the bulk field and accumulate on electrodes. The resulting double layer eventually screens the electric field in the bulk and restricts the full potential drop in immediate vicinity of the electrode-electrolyte interface. Bazant *et al*. [14] have provided convincing arguments that the rate at which the bulk field decays is characterized by time constant *t*_scr_ = λ*H*/*2D* where *D* is the diffusivity of the ions and λ (nm) is the Debye screening length, which at room temperature is related to the ionic strength of the medium, *IS* (M), by *IS* = 10.75λ^2^. The switching model of Bazant *et al*. is valid at voltages of order *V*_T_ = *kT*/*e* (*k* is Boltzmann’s constant, *T* absolute temperature, and *e* electron’s charge), which amounts to 26 mV at room temperature. In contrast, Beunis *et al.* [19] have offered a qualitative model that is applicable for arbitrarily high voltages.

**Figure 5:**
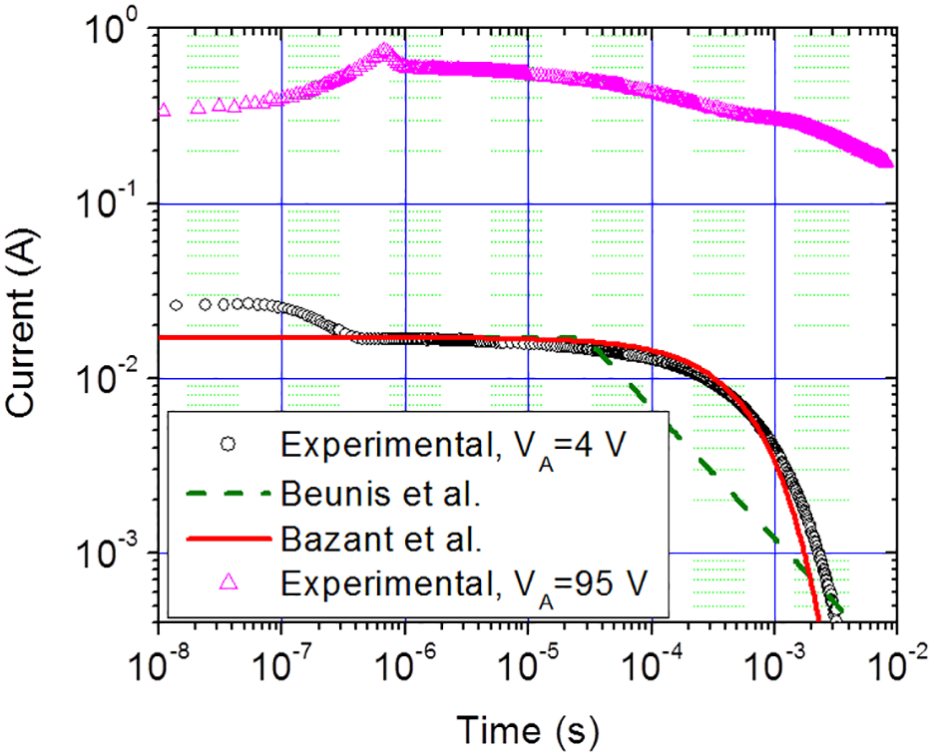
The electrical response of an electrical lysis chamber. An electrical lysis chamber, with dimensions 28(L)×6.4 mm(W)×0.1 mm(H), was filled with 0.25 mM NaCl solution and a fast rising step voltage with an amplitude of 4 and 95 volts was applied to the surface enhanced blocking electrodes. The response at the lower applied voltage was fitted to the models proposed by Bazant et al. [14] and Beunis et al. [19].

An E-Lysis chamber with dimensions 28 mm(L)×6.4 mm(W)×0.1 mm(H) was filled with 0.25 mM NaCl solution and a fast rising step voltage with an amplitude of either 4 or 95 Volts was applied, the electrical responses for which are shown in Figure 5. The response at the lower applied voltage (4 V) was fitted to the models proposed by Bazant *et al.* [14] and Beunis *et al.* [19]. The good agreement with the models which do not account for an electrode surface layer capacitance indicates that the presence of SEB electrodes does not appreciably reduce the switching response of the chamber in spite of the presence of a relatively thick dielectric (Al_2_O_3_) layer on the electrodes. The dielectric layer thickness *d* is about 140 nm since the thickness of the anodic Al_2_O_3_ layer is related to the formation voltage (100 V) by a proportionality factor of 1.4 nm/V [20]. In addition, the dielectric constant of Al_2_O_3_ layer is about 9 [21]. Therefore, without surface enhancement, these parameters would predict a capacitance C_DE_ = εε_0_ *WL*/*d* = 0.1 µF, with a corresponding R_CH_C_DE_ switching time of under 1.75×10^−5 ^s, a value 35 times shorter than the observed screening time for the SEB electrodes given in Figure 5.

The switching behaviour at the higher applied voltage (95 V) cannot be easily explained. The attempts to tackle the high voltage regimes [22]–[23] have not yielded satisfactory analytical approximations due to the onset of phenomena such as steric effects [24]. There is an indication from these studies that the charging time in high voltage regime should be shorter than its corresponding value at low voltage regime. However, this is not observed in Figure 5 where the screening time for the high applied voltage has increased by a large factor. This phenomenon could be due to the involvement of what may be identified as fractal surface charging. According to Sakaguchi and Baba [25] the effect of fractals on the electrical response can be accounted for by assuming that the temporal behaviour of current is given by *I* = *I*_0_exp(–(*t*/*t*_scr_)*^p^*) (*p*<1). In contrast, a qualitative inspection of Figure 5 shows that the temporal trend of the measured current is more akin to an exponential function of second order. We do not know how to explain this behaviour, but it should be noted that the longer charging time is advantageous for efficient cell lysis.

**Figure 6:**
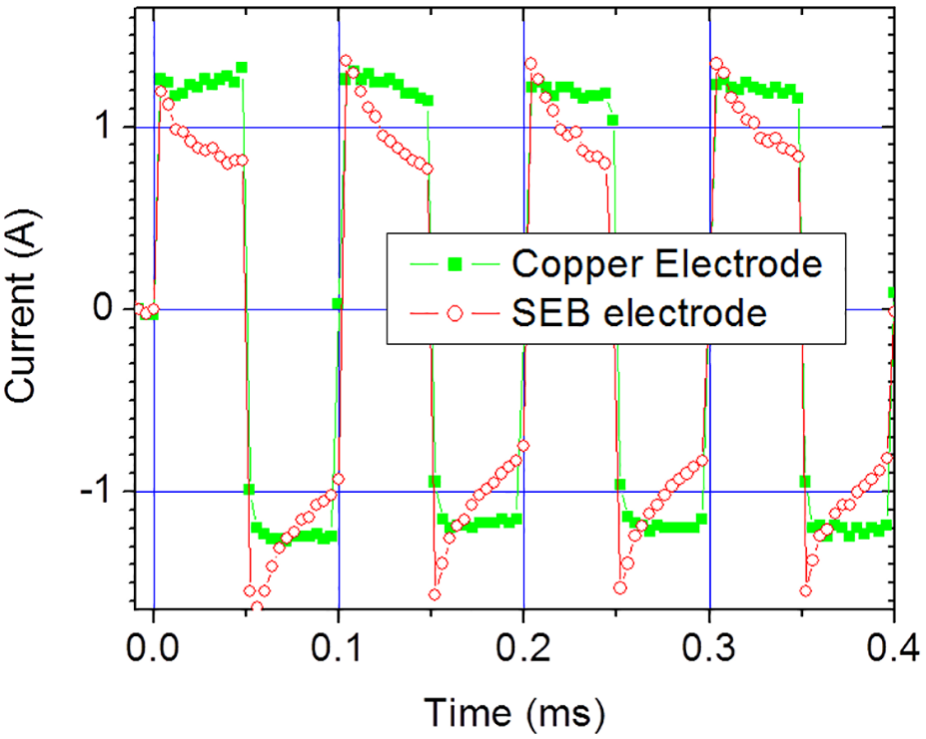
The effect of electrode type on current response of a lysis chamber. Two electrical lysis chambers, made respectively with copper and SEB electrodes, were filled with the microbial cell suspensions having ionic strength of 0.5

In order to demonstrate the blocking behaviour of SEB electrodes, two electrical chambers with dimensions of 15 mm(L)×6.4 mm(W)×0.2 mm(H) were fabricated using SEB and copper electrodes respectively. The chambers were filled with sodium phosphate buffer of ionic strength 0.5 mM and were driven by bipolar square pulses with amplitude of 195 V. The electric currents of two chambers are presented in Figure 6 for the first four cycles. As it is observed, in contrast to the case of copper electrode, there is an asymmetry in the charging and discharging behaviour for the SEB electrodes. The maximum amplitudes of the chamber currents during the charging cycle (positive current) are nearly identical for both cases. On the other hand, during the discharging cycle (negative current) the amplitude of current for SEB electrode is higher. This is due to the fact that SEB electrodes store electrical charge without electrochemical reactions at the electrode-electrolyte interface. The copper electrodes do not display this charging behaviour because of the presence of Faradic current involving electrolysis at the electrode-electrolyte interfaces.

### Thermal characteristics of the lysis device

When a voltage pulse train is applied to the chamber electrodes, the chamber fluid is exposed to an alternating electric field and substantial ionic current flows across the chamber causing rapid Joule heating. The peak temperature which can be reached by the fluid in the chamber is limited to the boiling temperature of the fluid, a property which is influenced by bubble nucleation conditions and pressure. For the case of chambers with ports open to atmospheric pressure, the maximum attainable temperature is approximately 100°C. The high heating rates of the electrical lysis device (on the order of 5000°C/s) may delay boiling by a few degrees [26]. However, the large surface to volume ratio of the chamber and the presence of numerous inhomogeneous bubble nucleation sites on the electrode surface may offset this effect, and therefore it is assumed herein that the onset of the liquid to vapour phase transition takes place at 100°C.

In general, the conductivity of an aqueous solution is a linearly increasing function of temperature [17] with a rate of 2–3% per °C, and therefore, when applying a pulse train the chamber current will rise due to Joule heating of the fluid in the chamber. Further energy input will result in the inception of a liquid to vapour phase transition in the region of the chamber which has reached this temperature. Subsequently the mixed phase in the central region undergoes rapid expansion, displacing the liquid out of the chamber ports. Accordingly, as the mixed phase region expands further, the ionic current within the chamber decreases substantially as net conductivity of the chamber falls. The displacement of the sample from the chamber is generally undesirable since it limits the exposure of the cell suspension to the electric field and elevated temperatures.

**Figure 7:**
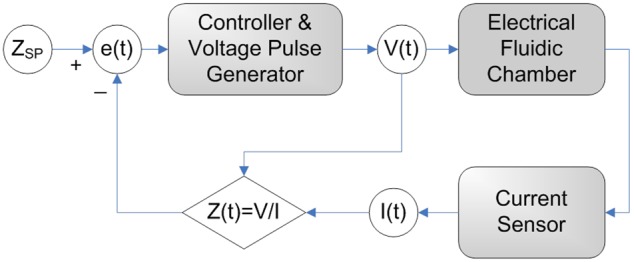
The block diagram of the impedance based temperature regulation scheme. The electric current supplied to the electrodes is measured and used as a proxy for the temperature of the chamber liquid. A feedback loop controls the voltage pulse train and maintains the chamber impedance at a pre-set value.

The lysis performance can be improved by controlling the voltage pulse train applied to the chamber so as to maximise the fluid temperature but prevent evaporation. The suspended cells are then exposed to the applied electric field and elevated temperature for an extended time. This requires shaping the pulse train by dynamically varying the pulsing rate or pulse amplitude with reference to a feedback signal related to the instantaneous temperature of the suspension medium. The E-Lysis device conveniently provides such a feedback signal from the current flowing across the chamber. Active feedback control of the voltage pulse train applied to the chamber is depicted by the control diagram shown in Figure 7. The temperature of the fluid in the chamber is indicated by the electrical impedance of the chamber, Z(t), which is obtained from measured voltage V(t) and current I(t). The instantaneous value of this impedance is used as the feedback signal. An error parameter e(t) is obtained from the impedance relative to a predetermined setpoint impedance Z_SP_ and provided to the controller. A wide range of choices exist for controlling the voltage pulse train in response to the error signal e(t). One example implementation is presented in the following paragraph.

**Figure 8:**
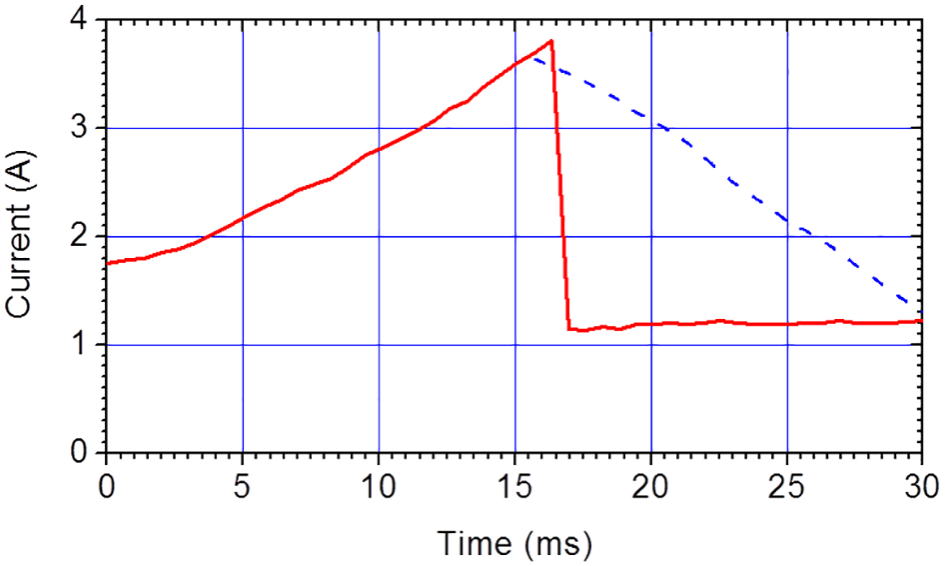
A typical current envelope of a lysis chamber operated under active feedback control of voltage pulse train amplitude. Feedback control of the voltage pulse amplitude in response to real-time chamber impedance measurements maintains the lysis chamber at a near constant temperature after reaching the setpoint at a time of about 16 ms. The dashed (blue) curve indicates the trend of current envelope in the absence of active feedback.

An initial voltage pulse train of constant amplitude and frequency is applied and upon reaching a predetermined chamber impedance setpoint, active feedback control is activated. Thereafter, the voltage controller rapidly lowers the voltage amplitude to a fraction of the initial amplitude and adjusts the amplitude of the remainder of the voltage pulse train in response to measured electrical impedance in order to maintain the setpoint impedance level. A typical current response corresponding to this scheme is presented in Figure 8 for initial voltage pulse train amplitude of 195 V and a frequency of 10 kHz. The setpoint current, chosen to be approximately equal to the peak current experienced by the same chamber for a constant voltage pulse train amplitude, was reached after t = 16 ms after which the voltage was adjusted downward to approximately 60 V. Thus a peak chamber temperature near 100°C is reached at the setpoint and as can be seen from the near constant current response, the temperature is approximately maintained at this level for the remainder of the voltage pulse train. Without this feedback control a liquid to gas phase transition would have occurred as represented by the dashed curve in Figure 8.

### Combined electric field and thermal lysis performance

**Figure 9:**
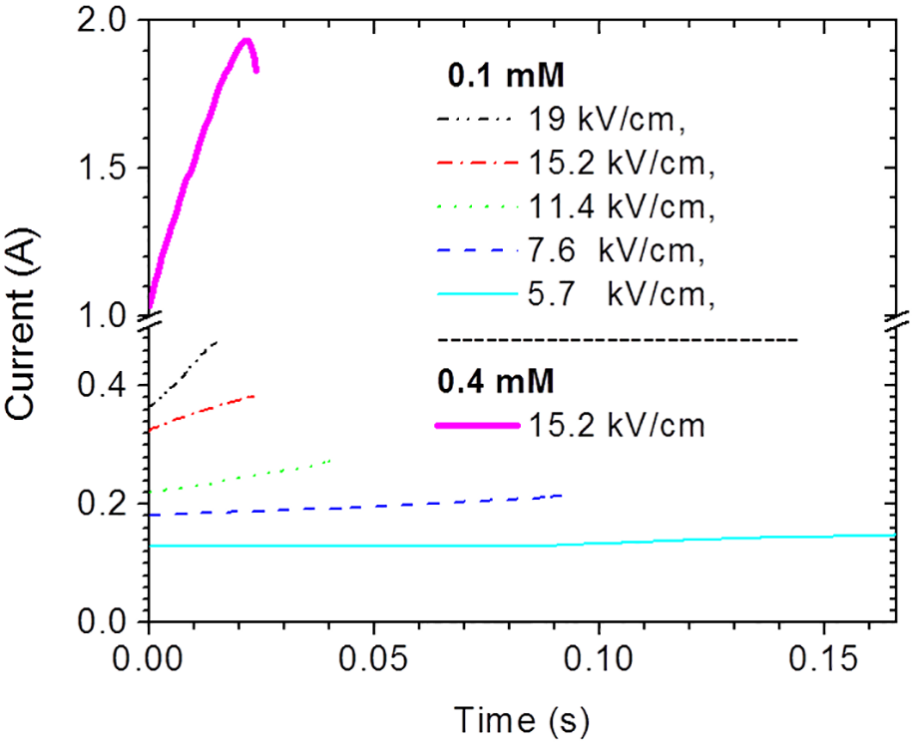
The current response envelope of lysis chambers operated under different applied voltages. Bipolar square pulses with different pulse amplitudes, *V*_A_, and pulse train durations, τ, were applied to a lysis chamber containing a cell suspension with ionic strength of 0.1 mM and the current envelopes were measured. Also presented is the current envelope for the case of *V*_A_ = 152 V applied to a cell suspension having ionic strength of 0.4 mM. The suspension in this case reaches boiling temperature prior to the end of the pulse train.

**Figure 10:**
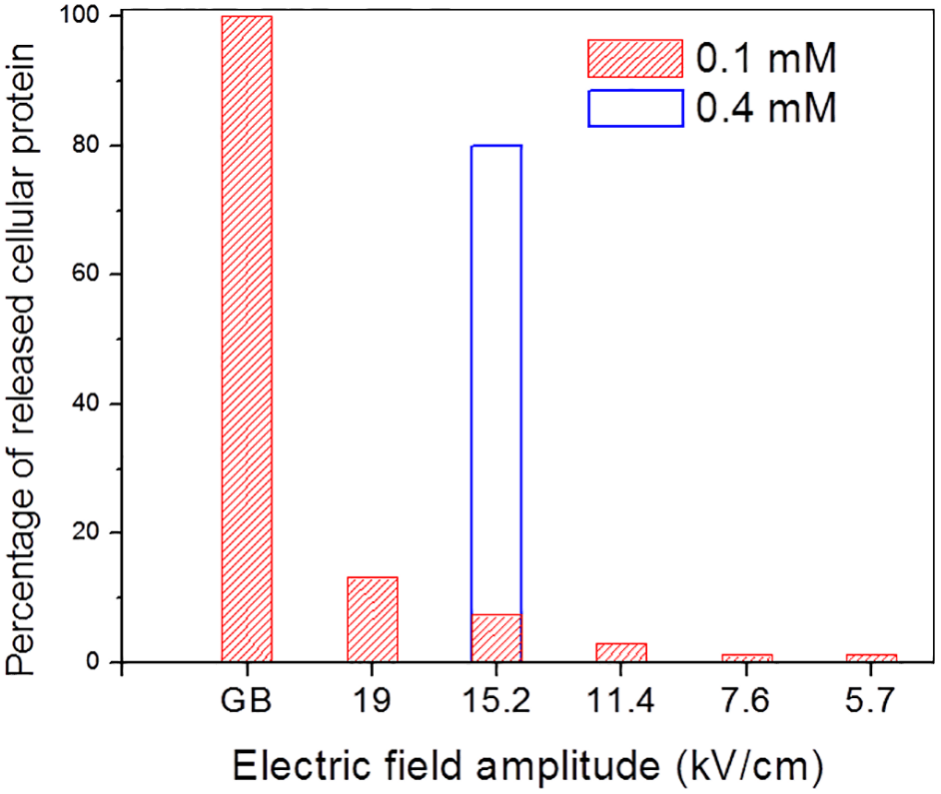
The lysis efficiency of *E. coli* as a function of electric field strength. The electrical lysis efficiency of *E. coli* cells for the cases presented in Figure 9 was determined by measuring the released protein as compared to glass bead beating. Only the case which involved flash heating gave comparable results indicating the synergy of electric field and heat lysis mechanisms.

The experiments described in this section are intended to demonstrate the synergistic effects of heat and electrical field on the efficiency of the device for lysing microbial cells. The performance was first demonstrated with Gram-negative bacteria *E. coli*. A bacterial suspension of 5×10^8^ CFU/mL in 0.1 mM sodium phosphate buffer pH 7.4 was lysed in an electrical lysis chamber, with dimensions of 28 mm(L)×3.2 mm(W)×0.1 (H)mm. The 10 kHz pulse train of duration τ consisted of 50 µs bipolar pulses with amplitude *V*_A_. The resulting electric field strengths across the chamber are indicated on Figure 9 which also shows the resulting current envelop. To ensure nearly equivalent electrical energy delivery to the chamber for purposes of comparison of lysis effectiveness, pulse train duration was chosen such that the quantity τ*V*_A_^2^ was kept approximately constant ( = 540 sV^2^) for all cases. Based on electrical conductivity changes, which are manifested by the rising currents in Figure 9, the temperature for all 0.1 mM cases increased a small amount, although not beyond the normal biological temperature range. The highest peak temperature reached was 42°C, which is obtained from the current rise for the applied field 19 kV/cm by applying the thermal conductivity factor of 2.5%/°C. This factor was obtained from the accompanying curve of the 0.4 mM suspension medium and applied field 15.2 kV/cm which reached boiling as indicated by the current drop near the end of the pulse train. A bacterial cell suspension of 5×10^8^ CFU/mL in 0.4 mM sodium phosphate buffer pH 7.4 was electrically lysed at an applied voltage of 152 V for a duration of 23.5 ms, electrically equivalent to the 15.2 kV/cm condition of the 0.1 mM case in Figure 9. However, due to the higher ionic strength the medium is rapidly heated and prior to the end of the pulse train the temperature reaches the boiling point. The results of a total protein assay are presented in Figure 10. Although the electric field amplitude is correlated to the amount of protein released from the cells during electrical lysis, the lysing effectiveness as judged by total protein release is much lower for electrical lysis using the 0.1 mM phosphate buffer as compared to GB lysis. By plating lysate on culture plates, we verified that no bacteria survived the injury inflicted by exposure to multiple electric pulses, as expected from published results [1], [3], [27]–[29]. However, the protein assay results indicate that employing an electric field alone for lysis of bacteria cells, with the goal of releasing macromolecules, is not an efficient procedure. However, when electrical lysis is combined with flash heating, as in the case for the example of the 15.2 kV/cm field in a 0.4 mM sodium phosphate buffer, the percentage of the released protein increased by over a factor of 10, and was about 80% as compared to GB lysis. Therefore, the simultaneous exposure of the bacteria to heat and electric field significantly enhances lysis efficiency.

**Figure 11:**
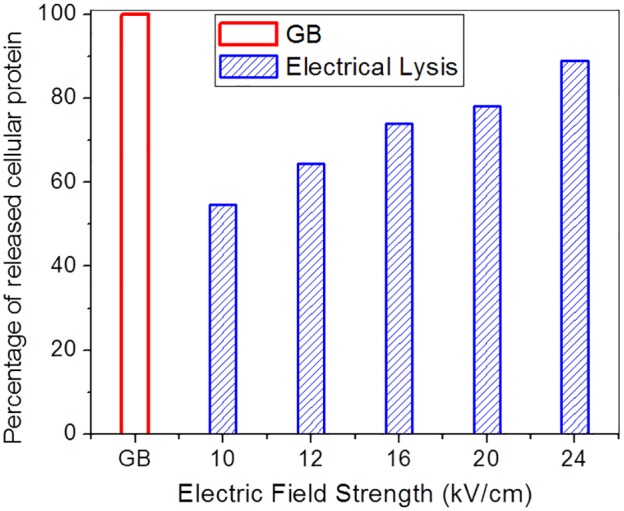
The lysis efficiency of *S. pneumoniae* as a function of electric field strength. The lysis efficiency of *S. pneumoniae* cells subjected to electrical lysis which combined electric field with flash heating to 100°C was determined by measuring the released protein. The amount of released protein is reported as a percentage of protein released by glass bead lysis.

The synergistic effect of the applied electric field and elevated temperature on cell lysis was further demonstrated with Gram-positive bacteria which are otherwise resistant to lysing by electric field alone due to their tough cell wall. The suspensions of 1×10^9^ CFU/mL *S. pneumoniae* in 0.4 mM sodium phosphate buffer pH 7.4 were lysed in a chamber with different parameter pairs (τ, *V*_A_) under nominally similar energy input defined by the quantity τ*V*_A_^2^ = 10^3^ sV^2^. In all cases the peak temperature momentarily reached 100°C prior to the end of the voltage pulse train. The results of total protein assay are presented in Figure 11, and it is observed that the lysis efficiency varies from 50% to over 80% for the range of electric field strengths tested.

### The effect of electrode type on lysis performance

**Figure 12:**
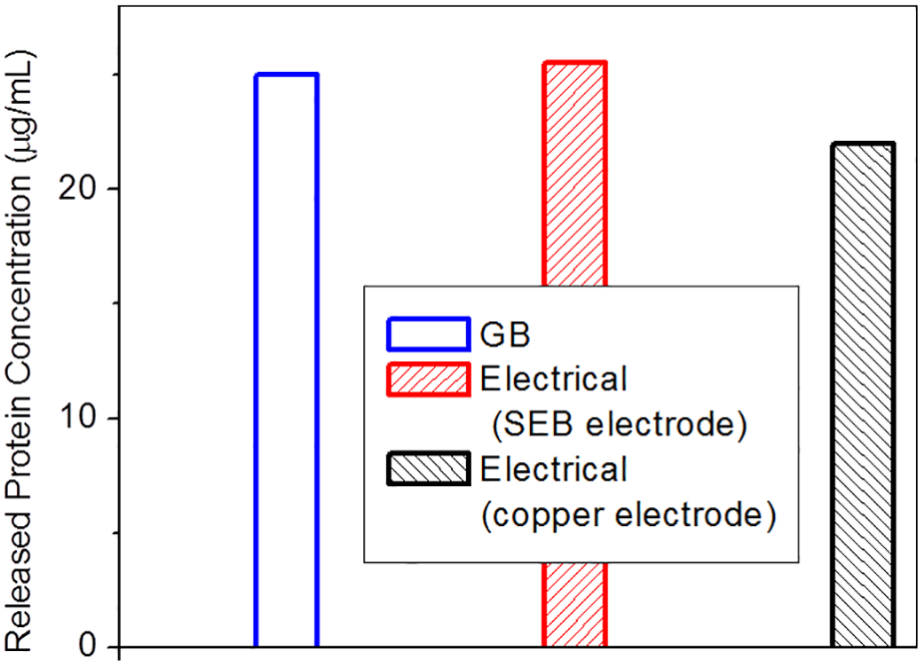
E-Lysis efficiency, with respect to protein release, in channels with SEB and copper electrodes. An electric field of 10/cm was applied until a peak temperature near 100°C was reached, followed by a constant temperature dwell with voltage feedback control. Both SEB and plain copper electrodes reach protein release concentrations similar to glass bead (GB) lysis.

The experiment described in this section is intended to demonstrate the advantages of using SEB electrodes for preserving the integrity of target macromolecules. *S. pneumoniae* cells, suspended in 0.5 mM sodium phosphate buffer pH 7.4, were lysed in two geometrically similar standard chambers, one chamber having SEB electrodes and the other having copper electrodes. The pulse amplitude was initially 195 V with feedback and a current response as shown in Figure 8. The corresponding electric field strength was about 10 kV/cm. The cell suspension intended for the Bradford protein assay and the real time RT-PCR assay respectively had a concentration of 1×10^9^ CFU/mL and CFU/mL. The result of protein assay is presented in Figure 12. The two chambers are similar in terms of protein release and their lysis performance is comparable to the GB beating. Therefore the electrode type does not affect lysis performance of the chambers for protein release.

**Figure 13:**
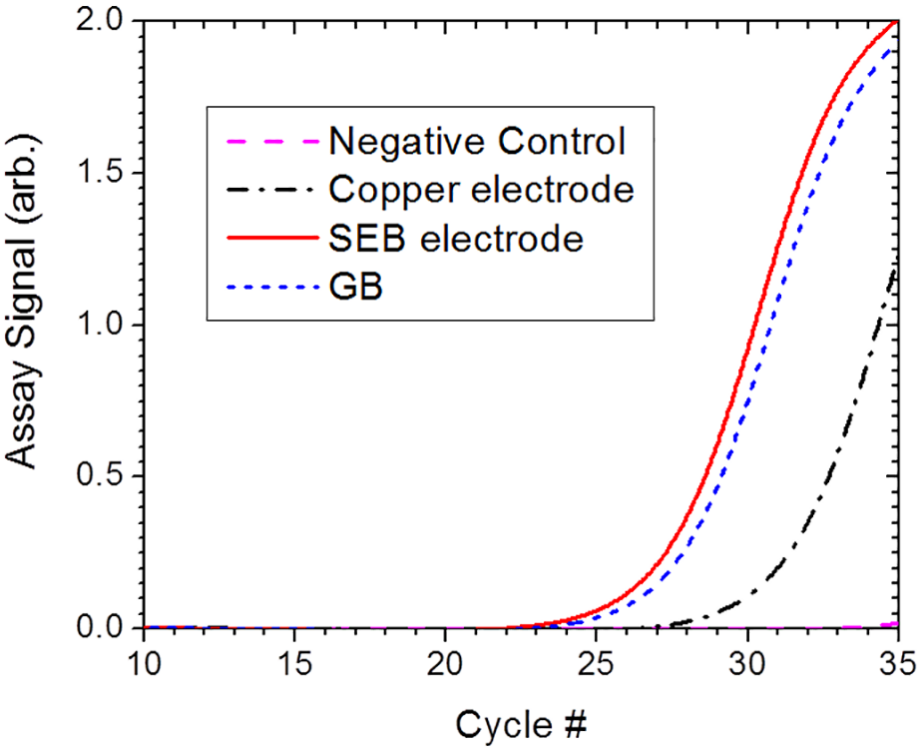
E-Lysis efficiency, with respect to nucleic acid assay, in channels with SEB and copper electrodes. The electrical lysis efficiencies of E-Lysis chambers using SEB and copper electrodes were compared by determining the amount of released ribosomal RNA from RT-PCR assay performed on the cell lysate.

With respect to performance in relation to nucleic acid assays the efficient release of molecular contents is not the only relevant criterion. The treated sample should not contain intolerable levels of PCR inhibitors and the target molecules must be intact. A real time RT-PCR assay was performed on the lysate of 200 CFU/mL cell suspension after being subjected to electrical lysis in chambers constructed with SEB and copper electrodes, respectively. The flash heating phase utilised a field strength of 10 kV/cm and voltage amplitude feedback control as above. A volume of 5 µL of the cell lysate, representing lysate from a single cell, was subjected to RT-PCR. The results, presented in Figure 13, indicate that the PCR amplification for the lysate from the chamber with SEB electrodes performs as well as GB lysis. The copper electrodes yield results which are delayed by 4 cycles relative to the SEB electrodes, implying an equivalent reduction in PCR products by a factor of 16. Since the protein assay result of Figure 12 suggests nearly similar macromolecule release efficiency, this implies that while using copper electrodes either the target nucleic acid has been degraded, possibly by the oxidizing species such as OH^−^ ions from the interfacial electrochemical reactions, or some ions released into the lysate are inhibiting the nucleic acid assay. In either case, the ability of SEB electrodes to provide assay-ready lysate is evident.

By monitoring the electrical current it was observed that the chamber made with copper electrodes degrades following application of pulse trains, indicated by a reduction in the current amplitude which could be achieved. After 10 actuations, the maximum current decreases by about half and the chamber may be judged as ineffective for lysing of volumes greater than approximately 50 µL. This is accompanied by discoloration of the initially polished surface which is a manifestation of an electrochemical reaction with the chamber fluid. In contrast, the chamber made from SEB electrodes is much more robust, not showing any evidence of degradation after lysing of as much as 100 mL of sample.

### Electrical lysing capability across different pathogenic bacteria

Further electrical lysing test results are presented to demonstrate the broad lysing capability of the device. Lysing was performed with the SEB electrode device for five pathogenic Gram-negative and five pathogenic Gram-positive bacteria species. Cell suspensions of each species were prepared at a concentration 200 CFU/ml in 0.5 mM sodium Phosphate buffer pH 7.4. A 60 µL each of cell suspension of each species and a 0.5 mM sodium phosphate buffer as a negative control was subjected to electrical lysis with an initial electric field of 10 kV/cm and voltage feedback control. RT-PCR was performed on 5 µL of lysate in each case. The detectability criterion was defined as follows. The standard deviation, σ, of the RT-PCR signal was calculated over the first 10 cycles, where the signal is predominately background noise. The cycle number for which the recorded real time PCR signal exceeds the threshold level of 6σ is defined as C_T_. If the C_T_ value of a sample differs by more than 5 cycles (a signal to noise ratio of over 32) from the C_T_ value of the corresponding negative control running in parallel with the sample, the detection is considered to be unambiguous.

**Figure 14:**
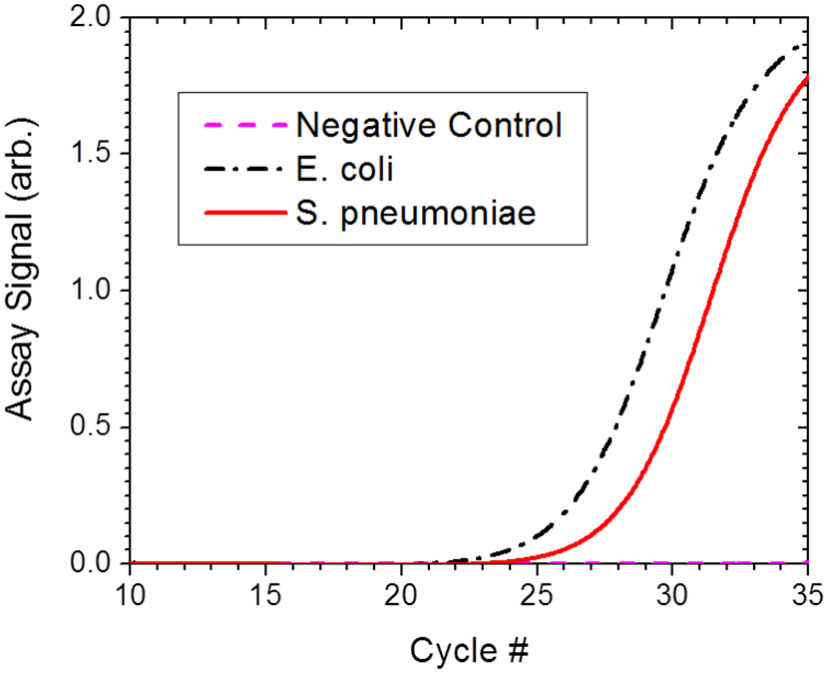
The real time RT-PCR results for detection of a variety of Gram-negative and Gram-positive bacterial species. Electrical lysis was demonstrated for a variety of Gram-negative and Gram-positive bacterial species using an E-Lysis chamber with combined electric field and flash heating to 100°C. This was demonstrated by performing real time RT-PCR assays directly on the lysate. Each data point represents 5 independent runs with two replica samples in each run.

The C_T_ values for the different Gram-negative and Gram-positive bacteria species over five independent runs, each run with two replicates, are presented in Figure 14. The detection limit criterion as described above was satisfied for all bacterial species samples tested. This demonstrates the effectiveness of E-Lysis as a rapid and reagent-free inline lysis method for sensitive detection of bacterial species.

**Figure 15:**
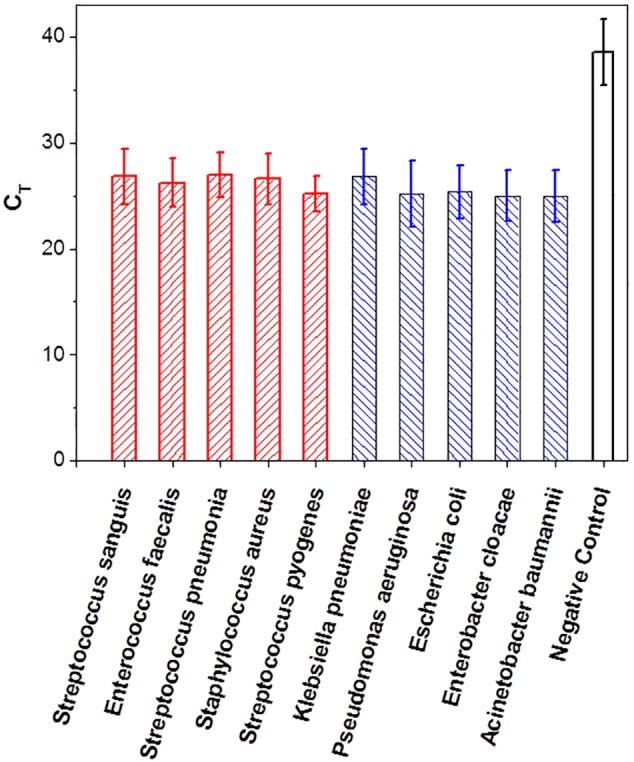
The electrical lysis efficiency of a polymicrobial sample. The performance of the electric lysis device for lysing a polymicrobial sample was demonstrated by lysing a cell suspension containing Gram-negative bacteria *E. coli* and Gram-positive bacteria *S. pneumoniae* cells and determining the amount of released ribosomal RNA from RT-PCR assay performed on the cell lysate.

The performance of the E-Lysis device was verified for the case of a polymicrobial sample. A cell suspension, containing 200 CFU/ml of Gram-negative bacteria *E. coli* and 200 CFU/ml of Gram-positive bacteria *S. pneumoniae* cells was prepared in 0.5 mM sodium phosphate buffer pH 7.4. A 60 µL aliquot of the suspension was electrically lysed. Simultaneous real time RT-PCR assays were performed on two 5 µL samples of the lysate in two separate wells, one containing Gram-negative bacterial specific primer set and the other containing Gram-positive bacterial specific primer set. The results are presented in Figure 15. Comparing the observed C_T_ values with the case of single microbial samples (Figure 14) indicated that the lysis performance of the device for a given bacterial species is not being influenced by the presence of other species in the sample. Therefore, the device is easily applicable in situations requiring multiplexed detection of bacterial species using the respective specific primers.

**Figure 16:**
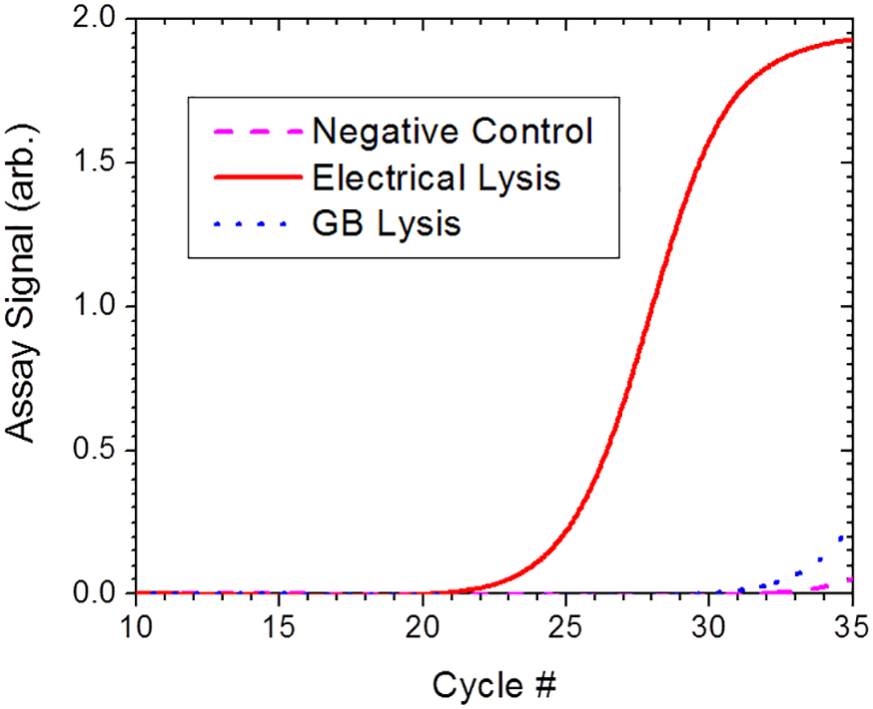
The electrical lysis efficiency of *Mycobacterium*. The lysis efficiency of *Mycobacterium smegmatis* cells subjected to electrical lysis which combined electric field with flash heating to 100°C was verified by determining the amount of released ribosomal RNA from RT-PCR assay performed on the cell lysate. The lysis efficiency of the electrical lysis method is higher than the lysis efficiency of glass bead beating technique by a factor of about 1000 (∼10 cycles).

Finally, the lysis performance of the E-Lysis device was tested for the *Mycobacterium* species, well known to be very difficult to lyse by conventional microbial lysis techniques. A cell suspension, containing 200 CFU/ml of the *Mycobacterium smegmatis* was prepared in 0.5 mM sodium phosphate buffer pH 7.4. A 60 µL aliquot of the suspension was subjected to electrical lysis using the E-Lysis device and another 60 µL aliquot was subjected to 5 minutes of glass bead beating as in previously discussed tests. Simultaneous real time RT-PCR assays were performed on 5 µL samples of the lysate in wells containing *Mycobacterium*-specific primer set. From the results presented in Figure 16 it is observed that the E-Lysis performance for the *Mycobacterium* is much more effective than the glass bead beating method used, yielding a C_T_ value about 10 cycles lower. Also, this result yields a C_T_ value similar to the values of the other bacteria species reported herein, indicating a similar lysis efficiency.

## Conclusions

Owing to the disadvantages of electrolysis from unprotected electrodes in microfluidic cell lysis devices, blocking electrodes are preferred for electrical lysis of microbial cells. However, the selection of blocking electrodes must mitigate the main drawback of such approach, namely the reduction in electrical screening time which can severely limit the effectiveness of electroporation by shielding the target cells from the applied electric field. This effect is largely eliminated by employing surface enhanced blocking electrodes with a finely micro-structured surface and a thin dielectric coating which increases the screening time to levels expected from bare electrodes. The use of such electrodes in thin fluidic chambers and the application of a bipolar voltage pulse train allow high electric fields of the order of 10 kV/cm to be generated in the cell suspension for a sufficient length of time to cause irreversible electroporation of cellular membranes. This configuration also causes rapid Joule heating from the ionic current generated in the cell suspension fluid and, by applying feedback control from measured electrical current across the chamber, temperatures in the neighbourhood of 100°C can be maintained during the pulse train. While electrical lysis of Gram-negative bacteria such as *E. coli* has been previously demonstrated, the current configuration and voltage regulation scheme provides a cell lysis device suitable for microfluidic applications and which can be used for a wide range of microorganisms, including Gram-positive bacteria and *Mycobacteria* whose cell envelope includes a tough cell wall. The effectiveness of this technique was demonstrated by lysing a range of pathogenic Gram-negative and Gram-positive bacterial species and directly using the lysate for performing real time RT-PCR without any further processing. The high performance of this lysis method is attributed to the synergistic effect of electrical field and high temperature on the bilayer membrane and cell wall.
