# Correction: Use of Surface Enhanced Blocking (SEB) Electrodes for Microbial Cell Lysis in Flow-Through Devices

**DOI:** 10.1371/journal.pone.0111288

**Published:** 2014-10-10

**Authors:** The PLOS ONE Staff

There is an error in the affiliation name. The correct affiliation should be: Qvella Corporation, Richmond Hill, Ontario, Canada.

The images for Figures 14 and 15 are incorrectly switched. The image that appears as Figure 14 should be Figure 15, and the image that appears as Figure 15 should be Figure 14. The figure legends appear in the correct order. Please see the Figures 14 and 15 in the correct order below.

**Figure 14:**
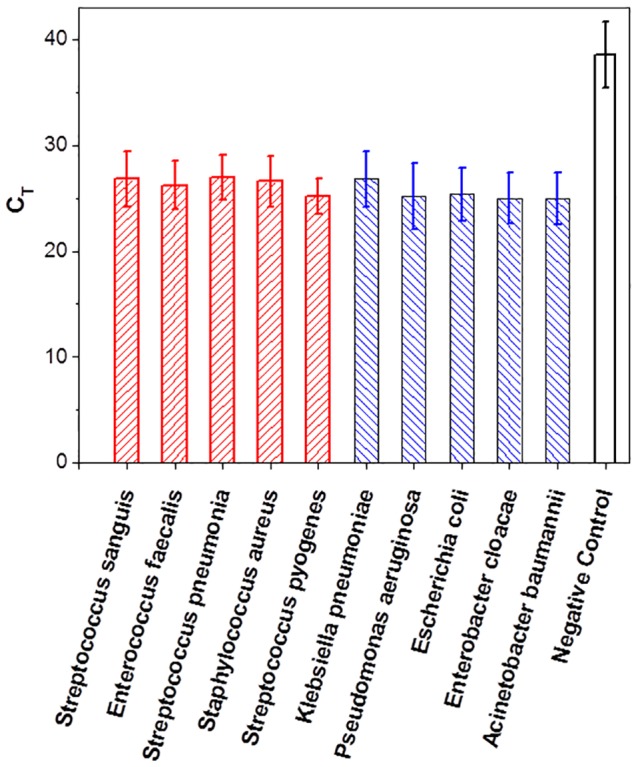
The real time RT-PCR results for detection of a variety of Gram-negative and Gram-positive bacterial species. Electrical lysis was demonstrated for a variety of Gram-negative and Gram-positive bacterial species using an E-Lysis chamber with combined electric field and flash heating to 100°C. This was demonstrated by performing real time RT-PCR assays directly on the lysate. Each data point represents 5 independent runs with two replica samples in each run.

**Figure 15:**
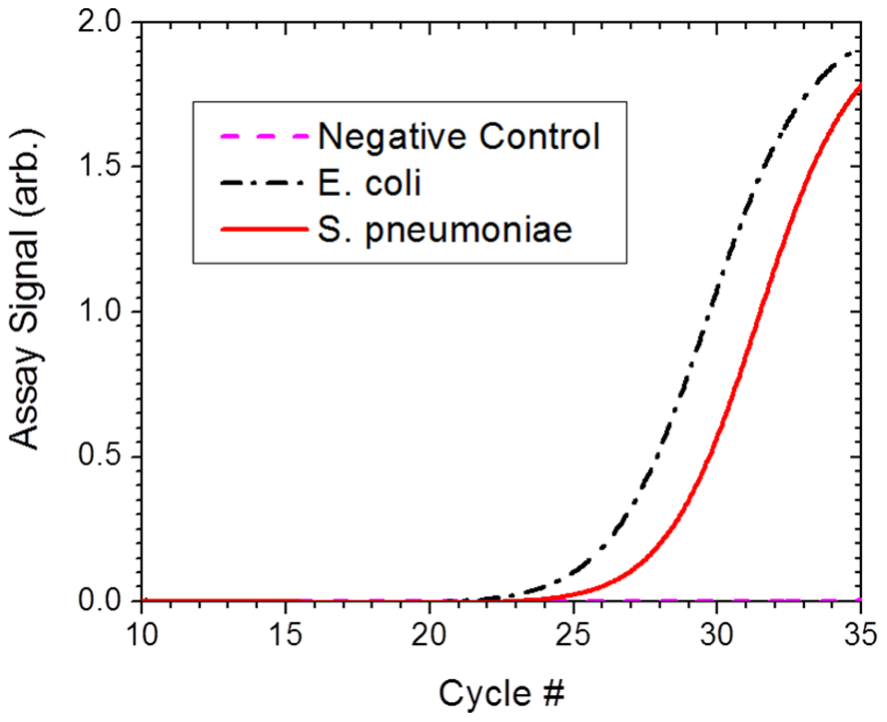
The electrical lysis efficiency of a polymicrobial sample. The performance of the electric lysis device for lysing a polymicrobial sample was demonstrated by lysing a cell suspension containing Gram-negative bacteria E. coli and Gram-positive bacteria S. pneumoniae cells and determining the amount of released ribosomal RNA from RT-PCR assay performed on the cell lysate.
